# Shitsu-taikan-sho (alexisomia): a historical review and its clinical importance

**DOI:** 10.1186/s13030-020-00193-9

**Published:** 2020-09-26

**Authors:** Takakazu Oka

**Affiliations:** Department of Psychosomatic Medicine, International University of Health and Welfare Narita Hospital, 852 Hatakeda, Narita, Chiba, 286-8520 Japan

**Keywords:** Shitsu-taikan-sho scale, Alexisomia, Alexithymia, Yujiro Ikemi, Interoception

## Abstract

“Shitsu-taikan-sho” is a clinical concept that refers to characteristics of having difficulty in the awareness and expression of somatic feelings or sensations. This concept was first proposed in 1979 by Dr. Yujiro Ikemi, the founder of psychosomatic medicine in Japan, as a characteristic observed in patients with psychosomatic diseases, i.e. physical diseases in which psychosocial factors are closely involved in their onset and progress. Soon after Dr. Ikemi introduced to Japan the concept of alexithymia, coined by P. E. Sifneos in 1973, he noticed that patients with psychosomatic diseases have difficulty in describing not only their emotions, but also somatic feelings and sensations. Dr. Ikemi proposed naming the concept of the trait of lacking somatic awareness “shitsu-taikan-sho” in Japanese (“alexisomia” in English), meaning “shitsu” a lack, “taikan” bodily feelings/sensations, and “sho” condition/symptoms. Dr. Ikemi observed characteristics of both alexithymia and alexisomia in patients with psychosomatic diseases, but considered alexisomia to have a more fundamental pathophysiological role in the understanding of psychosomatic diseases. He also emphasized the importance of treating alexisomia when treating psychosomatic diseases.

Recently, alexisomia has again come into focus for various reasons. One is the availability of the Shitsu-taikan-sho Scale (STSS), a self-rating questionnaire to evaluate alexisomic tendency. Another is recent advances in basic research on interoception. The former will facilitate clinical studies on alexisomia, and the latter will enable a deeper understanding of alexisomia.

This article is an overview of the historical development of the concept of alexisomia which was conceptualized by Dr. Ikemi, introduces the STSS, and discusses the current understanding and clinical importance of alexisomia in psychosomatic medicine.

## Introduction

“Shitsu-taikan-sho”, or alexisomia, is a clinical concept that refers to characteristics of having difficulty in the awareness and expression of bodily (somatic) feelings or sensations [1, 2]. This concept was first proposed in 1979 by Dr. Yujiro Ikemi, the founder of psychosomatic medicine in Japan, as a characteristic observed in patients with psychosomatic diseases [3], i.e. physical diseases in which psychosocial factors are closely involved in their onset and progress (the definition of psychosomatic disease by the Japanese Society of Psychosomatic Medicine, 1991).

In 1977, Dr. Ikemi introduced to Japan the concept of of alexithymia, a term coined by P. E. Sifneos [4]. At that time, Dr. Ikemi noticed that patients with psychosomatic diseases have difficulty in describing not only their emotions, i.e. alexithymia, but also somatic feelings/sensations. Later, he proposed naming the trait of lacking somatic awareness “shitsu-taikan-sho” in Japanese (“alexisomia” in English), meaning “shitsu” a lack, “taikan” somatic feelings/sensations, and “sho” condition/symptoms. Subsequent studies conducted in Japan suggested that there were patients with certain physical diseases who exhibited characteristics of alexisomia. These diseases include type 2 diabetes mellitus (T2DM) [5–7], bronchial asthma [8], peptic ulcer [9], and functional somatic syndrome [10].

Recently, the concept of alexisomia has again come into focus for two main reasons. One is the availability of the Shitsu-taikan-sho Scale (STSS), a self-rating questionnaire to evaluate alexisomic tendency [11, 12]. The scale will facilitate clinical studies on alexisomia. Another is the recent advances in basic research on interoception, any sense that arises from our inner body [13, 14]. Findings from interoception research will enable a deeper understanding of alexisomia.

This article is an overview of the historical development of the concept of alexisomia by Dr. Ikemi, introduces the STSS, and discusses the current understandings and clinical importance of alexisomia in the practice of psychosomatic medicine.

### Historical overview

Yujiro Ikemi (1915–1999) was the physician who established psychosomatic medicine in Japan. He founded the first Institute of Psychosomatic Medicine at Kyushu University in 1961 and from that time he saw many patients with psychosomatic diseases. He noticed early in his career as a psychosomatic medicine specialist that some patients with psychosomatic diseases manifested features of what was later coined “alexisomia.” However, he did not have an appropriate word to describe this characteristic and struggled with how to express it.

**Fig. 1:**
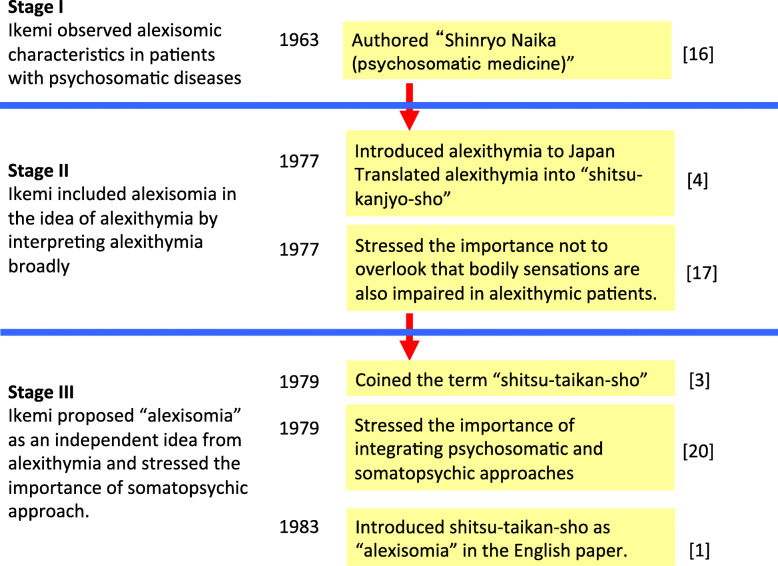
Historical timeline for the development of the concept of “shitsu-taikan-sho” (adapted from [15] with modification)

Here, we review the sequence of events leading Dr. Ikemi to establish and extend the concept of alexisomia. The timeline is divided into three stages (Fig. 1).

### Stage I: Dr. Ikemi noticed an alexisomic tendency in patients with psychosomatic diseases, but could not devise an appropriate term (1952–76)

Dr. Ikemi published articles regarding psychosomatic medicine from 1952, when he was at the Third Department of Internal Medicine at *Kyushu University*. In 1961, he became the first professor of the Research Institute of Psychosomatic Medicine, Faculty of Medicine, Kyushu University. In 1963, this institute was upgraded to a new department, the Department of Psychosomatic Medicine, and his clinical and research activities in psychosomatic medicine increased.

In 1963, Dr. Ikemi authored a book, “*Shinryo Naika*” (Psychosomatic Medicine), in which he explained the pathology and treatment of psychosomatic diseases through illustrations of cases and clearly explained the theories of that time regarding the mind–body relationship [16]. In this book, he introduced a female patient with obstinate rhinitis and stomatitis. Through the treatment of this patient, he discussed the cause of psychosomatic diseases. He stated, “In modern living, a functional gap can be formed between the cerebral neocortex, which busily responds to vast stimuli from the external world and the paleocortex, which responds to the demands of the internal world (instinct, impulse, and sensations from the internal organs). That is, modern living requires people to live in a condition where intellect and emotions are separate, as are mind and body”. At this time, Dr. Ikemi already had the idea that there were discrepancies between the intellect and emotions and between mind and body (sensations from the internal organ) in some patients with psychosomatic diseases, and that these discrepancies were at the core of the pathology. Dr. Ikemi likely had many such patients in his clinical practice. Therefore, it is easy to imagine that he was exploring a concept that accurately reflected such a state of discrepancies in patients with psychosomatic diseases.

However, his book triggered reactions that he did not intend. He stated afterwards that continuous inquiries came from all over Japan from patients with neurosis and near psychosis, but not from those with psychosomatic diseases, who were his target for treatment. Thus, at that time, he also looked for a concept that could distinguish psychosomatic disease from neurosis (neurotic disorder) and postulated several characteristics to distinguish the two. One characteristic was the mode of adaptation to the environment, i.e. “over adaptation” by patients with psychosomatic diseases, in contrast with the maladaptation of those with neuroses [17]. According to his theory, patients with psychosomatic diseases utilize excessive exertions to meet social demands (parent’s and teacher’s expectations of children, work achievements for adults, or being a “good wife” for women, i.e. over adaptation), which is associated with suppression of their own emotions (an idea close to “alexithymia”) and physical needs (what he later coined “alexisomia”). Such characteristics, i.e. putting higher priority on meeting social demands than following their own biological needs by suppressing the awareness of their emotions and physical needs made the patients good children, excellent workers, and good wives on the one hand, but frequently led to psychosomatic diseases on the other [17].

### Stage II: Dr. Ikemi included alexisomic tendency in alexithymia by broadly interpreting alexithymia (1977–78)

In 1973, Dr. Sifneos published the concept of alexithymia as a characteristic of patients with psychosomatic diseases [18]. He coined the word alexithymia to describe characteristics that include a relative constriction in emotional functioning, poverty of fantasy life, and an inability to find appropriate words to describe their emotions. He reported that the alexithymic characteristic was more prevalent among patients with psychosomatic diseases, including ulcerative colitis, bronchial asthma, peptic ulcer and rheumatoid arthritis, when compared with patients having neurotic complaints. He concluded that dynamic psychotherapy is not indicated for treating patients with psychosomatic disease because of their alexithymic characteristics [18].

In February 1977, Dr. Ikemi translated alexithymia into “shitsu-kanjyo-sho” in Japanese and first introduced this concept to Japan, with great reception because he realized that the presence and absence of an alexithymic tendency was very close to his idea about differentiating psychosomatic diseases and neurosis. He stated, “It is an important event that changes the conventional psychosomatic medicine, which has considered pathogenesis of psychosomatic diseases as the extension of neurosis and in which the main treatment approach has been psychoanalysis [4].” However, in July 1977 he added, “It is a problem that the idea of alexithymia exclusively focuses on suppression of emotional awareness. In people with a strong alexithymic tendency, it is important not to overlook the suppressed awareness of somatic feelings or sensations, which has a close relationship with awareness of emotions [17].” He also stated, “expansion of the definition of alexithymia and interpretation of the condition as ‘reduced awareness of emotions and body’ can provide a key to deepening the understanding of human diseases in general” and “the real purpose of psychosomatic therapy is the development of integrated, holistic understanding and self-control by intelligence based on awareness of the body and emotions [17]”.

These descriptions indicate that, for Dr. Ikemi, alexithymia was not necessarily a concept that described the characteristics of psychosomatic diseases in a satisfactory manner. However, at the time, there was no other appropriate concept (such as alexisomia) that could be used as a replacement. Therefore, he attempted to express the characteristics of psychosomatic diseases by borrowing the concept of Dr. Sifneos and interpreting alexithymia broadly with explanatory remarks [19].

### Stage III: Dr. Ikemi coined “shitsu-taikan-sho” or “alexisomia” as a concept independent from alexithymia (1979–)

In 1979, Dr. Ikemi first used the term “shitsu-taikan-sho” in the foreword of “*Kouryubunseki Kenkyu*” (Transactional Analysis Research) [3]. He stated, “In the era of rapid economic growth, the objective of the current educational boom seems to create prodigies and elite employees, who can adapt to the artificial society through training of intellectual self-efficiency. As this trend gets stronger, conditions such as alexithymia (I include “shitsu-taikan-sho” in this concept), which has become a problem in the field of psychosomatic medicine in recent years, and ‘anti-naturalism’ have become the root of not only modern diseases but also a general crisis of the modern era [3]”. He also explained, “we observe that many of the modern diseases, especially adult diseases, are based on the diminished awareness of bodily feelings that is balanced with eating habits and exercises that form the basis of body homeostasis. Blunted awareness of the body also leads to impairment of the laws of nature that are directly connected to the body [3]”. In 1979, Dr. Ikemi proposed integrated psychosomatic treatments by using the word “interoceptive awareness”, in which he stated the primary importance of restoring an optimal level of interoceptive awareness was so that the patient can learn prohomeostatic self-control [20].

In 1983, Dr. Ikemi translated “shitsu-taikan-sho” into alexisomia in English and first introduced this concept in an English language article [1]. He explained alexisomia as follows, “in many cases of ‘alexithymia’, where there is an observed difficulty in the awareness and expression of feelings, there also seems to be a difficulty in the awareness and expression of bodily feelings. We have tentatively coined the term ‘alexisomia’ to designate this condition, where certain persons have difficulties in expressing how their bodies feel” [21]. Soon after proposing alexisomia independently of alexithymia, Dr. Ikemi began to think that alexisomia plays a more fundamental pathophysiological role in the development of psychosomatic disease [22].

Furthermore, he proposed that body-oriented, somatopsychic approaches be introduced as a treatment of psychosomatic diseases, which included biofeedback, focusing, autogenic training, yoga, za-Zen (Zen Buddhist style meditation), or a self-regulation method he developed [23]. As many of these techniques had been practiced in the East, he proposed the importance of integrating an “occidental”, psychosomatic approach, in which the mainstream of the treatment was verbal psychotherapy, and an “Oriental”, somatopsychic approach, which aims to restore an optimal level of interoceptive awareness, as a holistic and integrative therapeutic model of psychosomatic diseases [17, 20, 21, 24–26].

### Development of the Shitsu-taikan-sho scale

After proposing the concept of alexisomia, Dr. Ikemi provided numerous descriptions of alexisomia (Table 1) (for review, see [27]). Subsequent observational studies indicated that there were patients with psychosomatic diseases who manifested alexisomic characteristics, including those with T2DM [5–7], bronchial asthma [8], peptic ulcer [9], and functional somatic syndrome [10] (for review, see [28]). However, there was no tool, especially a self-rating tool, with which to assess alexisomia until 2012, when we developed the Shitsu-taikan-sho Scale (STSS) [11, 12, 29]. This was in contrast with the many self-rating questionnaires that have been developed to assess alexithymia [30–32]. Among them, the 20-item Toronto Alexithymia Scale (TAS-20) [31, 32] has been used widely in Japan [33]. The TAS-20 has three subscales: difficulty in identifying feelings, difficulty in describing feelings, and externally oriented thinking [31, 32].

**Table 1:** Representative descriptions on shitsu-taikan-sho (alexisomia) and its related ideas by Yujiro Ikemi

1. We have repeatedly noticed that so-called alexithymic features exist together with disturbances in awareness of body sensations. For example, girls suffering from anorexia nervosa do not seem to know what fatigue is (1977) [19].	
2. Recently, an interesting theory has been advocated that the neurophysiological dissociation of neocortical intellectual functions and subcortical emotional functions may be an important feature of the basic pathology of psychosomatic disorders. This state has been called “alexithymia”. According to our observations, this dissociation involves not only the awareness of emotions, but also awareness of body sensation. Emotion, which is a neurophysiologic phenomenon (produced through “emotional circuits”) is closely related to bodily sensation, and can be created and altered by physiologic procedures (1979) [20].	
3. In the state of cortical hyperactivity (ergotropic dominance) and inhibition of subcortical activity, ego-related feedback of different kinds may develop which replaces the normal proprioceptive afferent feedback from the body itself. This process may contribute to the formation of alexithymic state. From this point of view somatopsychic approaches may play an important therapeutic role in treating the so-called alexithymic state, or psychosomatic conditions where the dissociation between ego functions and emotion (feelings) is playing the predominant role (1979) [20].	
4. One is the primary importance of restoring an optimal level of interoceptive awareness to the patient so that he, or rather his body, can learn prohomeostatic self-control. For this purpose the somatopsychic orientation is indispensable, and we envision it becoming an essential basis of both psychosomatic and general clinical medicine (1979) [20].	
5. Alexisomia refers to the disturbance in one’s bodily sensations with psychic prevalence of the alexithymic features. Ikemi and Ikemi argued that patients with alexithymia also have this alexisomic defect (1985) [6].	
6.In many cases of ‘alexithymia’, where there is an observed difficulty in the awareness and expression of feelings, there also seems to be a difficulty in the awareness and expression of bodily feelings. We have tentatively coined the term ‘alexisomia’ to designate this condition where certain persons have difficulties in expressing how their bodies feel (1986) [21].	
7.I wish to focus my speech to the holistic awareness which can be the root of holistic medicine and the root of Oriental somatopsychic self-control which plays an essential part in the enlargement of experiential level by the dissolution of alexithymia, alexisomia and alexicosmia. Soon after I introduced Prof. Sifneos’s concept of “alexithymia” to Japan, I noticed that in many cases of alexithymia there also seemed to be a difficulty in the awareness and expression of bodily feelings and the natural order implicit in the body. I have coined the term “alexisomia” to designate a condition, where certain persons have difficulty in expressing how their bodies feel, and the term “alexicosmia” to designate a condition marked by a lack of awareness to the natural order (1990) [2].	

The STSS is a 23-item self-rating questionnaire to assess alexisomia. Initially, we developed a 44-question item draft scale based upon Dr. Ikemi’s descriptions of alexisomia [27]. Exploratory factor analyses resulted in a 23-item instrument with an adequate oblique 3-factor structure. With reference to the TAS-20 and Dr. Ikemi’s descriptions, we named these subscales: (1) difficulty of identifying bodily feelings (DIB); (2) over adaptation (OA); and (3) lack of health management based on bodily feelings (LHM) [12] (Table 2). (1) The DIB subscale consists of questions asking about a tendency to fail to notice sensations that act as warning signals emerging from the body during adaptation to external environments and that are necessary to maintain the body’s homeostasis (such as hunger, tension, and fatigue), and failure to notice relaxed feelings that are needed to prevent allostatic load. (2) The OA subscale consists of questions inquiring into a tendency to ignore warning signals from the body that result from prioritizing meeting social demands and adapting to external environments despite a feeling of fever, fatigue, sleepiness, feeling unwell, or a desire to rest. This subscale reflects Dr. Ikemi’s idea that over adaptation to environments created by modern society is associated with suppressed awareness of homeostatic somatic feelings [17, 35]. (3) The LHM subscale consists of questions inquiring into habits related to managing health on a daily basis and bodily sensations coming from physical conditions resulting from the relaxation response.

**Table 2:**
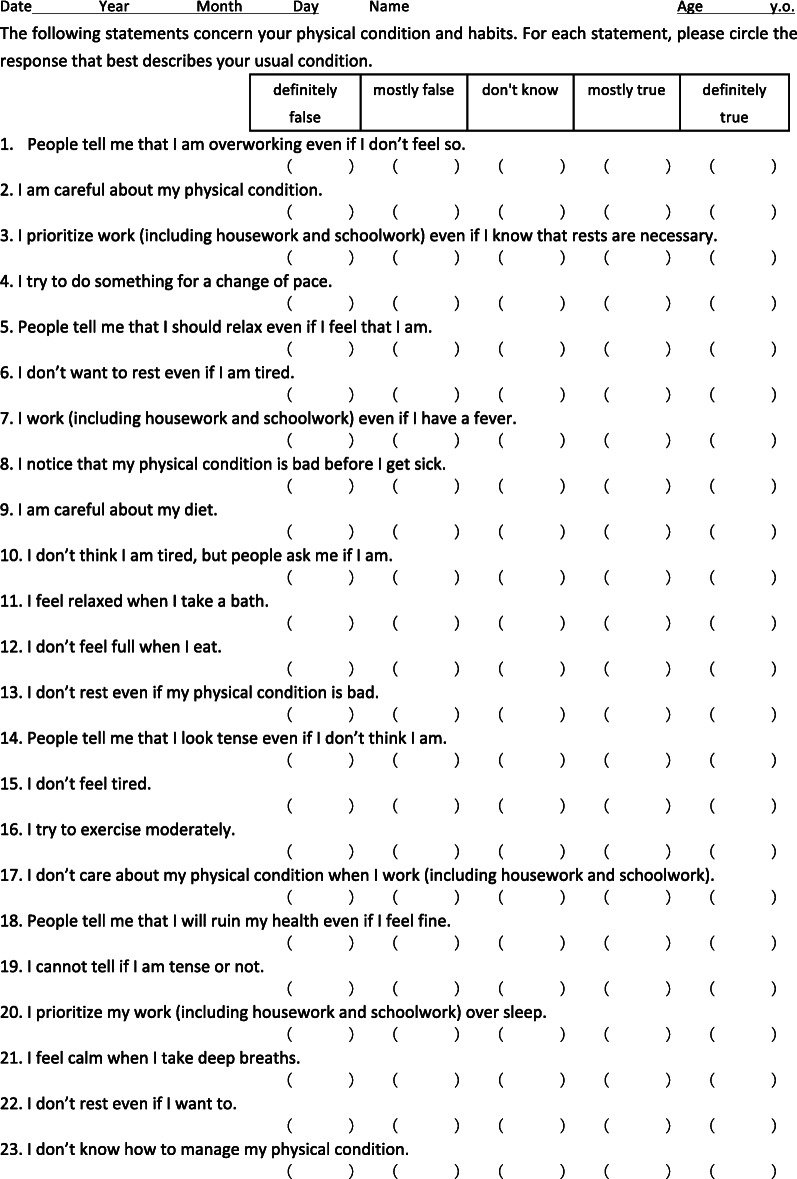
Shitsu-taikan-sho scale (STSS)

The STSS is a 23-item self-rating questionnaire to assess alexisomia and has 3 subscales: (1) Difficulty of identifying bodily feelings (DIB); (2) Over adaptation (OA); and (3) Lack of health management based on bodily feelings (LHM). The questions are rated using a 5-level Likert scale and the total score was 115. (1) DIB is made up of questions 1, 5, 6, 10, 12, 14, 15, 18, and 19, and the among Japanese college students was 18.1 ± 5.6 (average score ± standard deviation) points. (2) OA is made up of questions 3, 7, 13, 17, 20, and 22, and the average score was 14.2 ± 4.5 points. (3) LHM is made up of 8 items, namely questions 2, 4, 8, 9, 11, 16, 21, and 23. Only 23 is regular scoring, but all the others are reversely scored items. The average score was 21.4 ± 3.8 points. Thus, the total average score was 56.3 ± 10.2 points (of 115 total). The original version was constructed in Japanese [11], which is downloadable [34]. In this table, an English translated version is shown

As is expected, the STSS total and subscale scores have been demonstrated to have strong associations with the TAS-20 total and subscale scores of college students [11] and middle-aged women [36] (Table 3). In both studies, the STSS total score has a positive correlation with TAS-20 total score and TAS-20 difficulty in identifying feelings subscale score. Among their subscales, a strong correlation is found between the STSS difficulty of identifying bodily feelings subscale score and TAS-20 difficulty in identifying feelings subscale score [11, 36]. Furthermore, as Dr. Ikemi had predicted, several case reports have indicated that regular practice of yoga improved alexisomia, which was evaluated by doctors or psychotherapists, in accordance with the reduction of the STSS score and with improvement of the symptoms of patients with eating disorders [37], primary headache, psychogenic fever and hypertension [38], and myalgic encephalomyelitis/chronic fatigue syndrome [39].

**Table 3:** Correlation between STSS total and subscale scores and TAS-20 total and subscale scores (cited from [11])

	TAS-20 Difficulty identifying feelings	Difficulty describing feelings	External oriented thinking	Total
STSS				
1. Difficulty of identifying bodily feelings	0.45***	0.21**	0.19**	0.43***
2. Over-adaptation	0.28***	0.11	0.09	0.25***
3. Lack of health management based on bodily feelings	0.17*	0.19**	0.14	0.24**
Total	0.44***	0.24**	0.20**	0.45***

The strongest correlation was found between the STSS difficulty identifying bodily feelings subscale and TAS-20 difficulty identifying feeling subscale. ***p < 0.001, **p < 0.01, *p < 0.05

The STSS was originally constructed in Japanese and its English translation is shown in Table 2. The original Japanese version of the STSS can be downloaded from the internet [34].

### Clinical importance of alexisomia in psychosomatic medicine

As described above, alexisomia is a clinical concept through which Dr. Ikemi aimed to describe the characteristics he observed in patients with psychosomatic diseases. To obtain a better understanding of the role of alexisomia in psychosomatic diseases, I would like to introduce a case report by Dr. Nobuo Kurokawa, one of Dr. Ikemi’s disciples, who described his clinical experience of treating a patient with T2DM whose glycemic control was very poor [7]. The patient was a man in his 50s who had been treated with medication. Despite poor glycemic control, the patient was indifferent to the disease and refused hospitalization because he claimed he felt that nothing was wrong with him (i.e. alexisomia), even though his blood sugar level became almost 1000 mg/dL due to an infectious disease. Psychiatric evaluations revealed that the patient was not depressed and his anxiety level was very low. However, through conversations with the patient, Dr. Kurokawa noticed that the patient had characteristics of alexithymia. As part of his treatment, Dr. Kurokawa asked the patient to predict his blood sugar level on every visit to the clinic. At first, the patient could not predict the level, even approximately. However, as he began to be mindful of the feelings arising from his body and to learn discrepancies between the predicted value and the true value, he was able to predict his blood sugar level almost correctly as he began to feel various physical discomforts, depending on his blood sugar level. Subsequently, his glycemic control improved. Later, the patient stated, “When my blood sugar was around 1000 mg/dl, I should have felt more fatigued than now. It is horrible that I didn’t feel anything wrong at that time [7].” Through this intervention, he not only became less alexithymic and less alexisomic, but also came to feel physical symptoms that many T2DM patients have. Furthermore, he became able to face his illness and had restored a sense of well-being. Cumulative experiences like this case must have convinced Dr. Ikemi of the idea that alexisomia is a fundamental characteristic of patients with psychosomatic diseases [22]. Previous studies in the field of psychosomatic medicine have identified numerous psychosocial factors that can affect the onset and progression of physical diseases. These include socioeconomic status, stressful life events, adverse childhood experiences, comorbid psychiatric disorders, and characteristics of the affective (e.g. fear and anxiety), cognitive (e.g. catastrophizing), or behavioral (e.g. health-seeking, type A) dimensions, as well as alexithymia. However, to date, too little attention is paid to alexisomia, despite the author having encountered a considerable number of patients like this one in the practice of psychosomatic medicine. This case report clearly indicates that alexisomia was associated with poor control of T2DM and that its treatment brought a beneficial clinical outcome. This highlights that alexisomia would be worth studying as a factor in the onset and/or the progress of psychosomatic diseases. The STSS would be a helpful tool for conducting clinical studies of the role of alexisomia in psychosomatic medicine.

### Differences from somatic symptoms measured by the TAS-20

When considering research on alexisomia, it is important to keep in mind that the STSS measures different dimensions of “bodily feelings” from the TAS-20. Originally, Dr. Sifneos did not use any questions related to somatic feelings in the Beth Israel Hospital (Boston) psychosomatic questionnaire, which he developed to determine alexithymic characteristics [18]. However, self-rating questionnaires that were developed later used question items asking about physical symptoms and related feelings to evaluate alexithymia. For example, the 22-item Minnesota Multiphasic Personality Inventory–Alexithymia Scale (MMPI-AS) includes questions such as “I am troubled by discomfort in the pit of my stomach every few days or more often” and “I have to urinate no more often than others” as questions used to evaluate alexithymia [30]. The TAS-20 also includes questions such as “I have physical sensations that even doctors don’t understand (Q3),” and “I am often puzzled by sensations in my body (Q7),” as questions to assess the difficulty identifying feelings subscale of alexithymia [31]. Kleiger and Kinsman, who developed the MMPI-AS, interpreted these items as reflecting an alexithymic subject’s preoccupation with somatic cues [30] and that this interpretation may also be the case with items in the TAS-20. By contrast, as indicated in the case report, somatic feelings where alexisomia is concerned are those that act as a warning sign to prevent worsening of the disease and to help recover health, which Dr. Ikemi called the “wisdom of the body” [20]. He thought that awareness of such somatic feelings is impaired in some patients with psychosomatic diseases and that, therefore, facilitating awareness of these feelings brings about better clinical outcomes.

Thus, impaired awareness of somatic feelings that have recuperative significance, which is measured by the STSS, is different from physical symptoms that alexithymic individuals are preoccupied with and complain about, which are measured by the TAS-20. In other words, there may be two different types of somatic feelings in alexithymic individuals, impaired/unaware somatic feelings and those that the individual is preoccupied with or aware of. Dr. Ikemi focused on the former, which the STSS measures, and the TAS-20 measures the latter.

### Alexisomia: just an impaired interoceptive awareness?

Currently, the term “bodily feelings” that Dr. Ikemi referred to can be replaced by interoception, the sense of the physiological condition of the entire body [13]. Therefore, alexisomia could be described as impaired interoceptive awareness [3, 20]. Interestingly, recent studies have suggested that alexithymia is associated with impaired interoception [40–43]. Furthermore, several studies have shown that impairment is not restricted to only one domain, e.g. cardiac, but is seen in multiple domains, such as respiratory domains, in alexithymic individuals [44, 45]. Therefore, some investigators claim that alexithymia is not merely a cognitive and affective deficit, but rather characterized by a general impairment of interoception [44–48]. In contrast, other studies have drawn a contradictory conclusion, viz., that alexithymia is associated with increased interoceptive awareness and amplified bodily sensation [49–51].

These conflicting results might simply reflect different somatic manifestations in alexithymia, as was discussed in the “Differences from somatic symptoms measured by TAS-20” section. Or, they might come from the complexity of interoception and the differences in experimental protocols used to measure it. Interoception is involved in many different systems, such as cardiac, respiratory, gastrointestinal, genitourinary, nociceptive, thermoregulatory, endocrine, and immune systems. It also encompasses multiple neural processes, including not only afferent signaling, but also neural encoding, representation, and integration of the information concerning the internal bodily state; the influence of such information on other perceptions, cognitions, and behaviors; and expression of these representations as consciously accessible physical sensations and feelings [52]. From another perspective, interoception has various dimensions, i.e. interoceptive accuracy (sensitivity), interoceptive sensibility, interoceptive awareness, and others [14, 53, 54]. Therefore, it is uncertain whether the conclusions drawn from experiments using one specific method, e.g. heartbeat detection task to measure cardiac interoceptive accuracy, can generalize to characteristics of interoception in other systems and dimensions, alexithymia, or alexisomia.

When considering the case of T2DM, individuals with alexisomia may be those who have difficulty in the awareness of disease-related interoception, such as thirst and fatigue, in the first instance. Furthermore, there are also those who do not regard such interoceptive warning signs as important, or ignore them, and those who do not use appropriate coping behaviors. Dr. Ikemi called such therapeutic interoception the “wisdom of the body” and stressed the importance of restoring an optimal level of interoceptive awareness and learning prohomeostatic self-control when treating psychosomatic diseases [20]. Thus, alexisomia may not simply be an awareness of interoception that comes from one specific system, but rather a constellation that consists of multiple systems, multiple neural substrates, and multiple dimensions. To describe alexisomia more appropriately when using the concept of interoception, further studies are warranted.

There are several limitations to this work. The idea of alexisomia was developed from Dr. Ikemi’s clinical experience in Japan. Therefore, the idea of alexisomia and the question items of the STSS apply to the Japanese context and culture. Furthermore, descriptions of the possible role of alexisomia in psychosomatic medicine are based on observational studies conducted exclusively in Japan, as there has been no study on alexisomia outside Japan and there are to date no cross-cultural studies on alexisomia. Further studies are required to draw conclusions about the role and importance of alexisomia in other cultural settings.

In addition, more comprehensive studies are necessary to determine if the STSS correctly measures alexisomia. Recent studies suggest that there are different subgroups of individuals with high TAS-20 scores [55]. Therefore, researchers recommend using TAS-20 as a rough indicator of alexithymia rather than as a one-dimensional measure of alexithymia [55]. Similar studies are warranted for the STSS.

## Conclusions

This article is an overview of the development of the concept of alexisomia by Dr. Yujiro Ikemi and discusses the clinical importance of alexisomia in psychosomatic medicine. It is to our surprise that, as early as the 1970s, Dr. Ikemi already had an idea of alexisomia as a pathophysiological characteristic of psychosomatic disease and proposed the importance of restoring an optimal level of interoceptive awareness for treating psychosomatic illnesses. To obtain a deeper understanding of alexisomia and to establish the clinical importance of alexisomia in psychosomatic medicine, further studies, including experimental, clinical, and cross-cultural studies, are necessary.

## Data Availability

No data sets were generated or analyzed during the present study.

## Abbreviations

DIB: Difficulty of identifying bodily feelings
LHM: Lack of health management based on bodily feelings
MMPI-AS: Minnesota Multiphasic Personality Inventory–Alexithymia Scale
OA: Over adaptation
STSS: Shitsu-taikan-sho Scale
TAS-20: Toronto Alexithymia Scale-20
T2DM: Type 2 diabetes mellitus
